# Necrotizing Pancreatitis Secondary to Hydrochlorothiazide and Alogliptin: A Case Report

**DOI:** 10.7759/cureus.37754

**Published:** 2023-04-18

**Authors:** Teresa Del Rio, Manveer Ubhi, Luis E Irizarry Nieves, Basilides Fermin, Kala Sury

**Affiliations:** 1 Internal Medicine, Wyckoff Heights Medical Center, Brooklyn, USA; 2 Pulmonary Critical Care, Internal Medicine, Wyckoff Heights Medical Center, Brooklyn, USA

**Keywords:** necrotizing pancreatitis complication, medication-induced pancreatitis, medication induced, acute necrotizing pancreatitis, severe pancreatitis

## Abstract

Drug-induced pancreatitis occurs rarely but should be considered when more common causes have been ruled out. While simple to treat, mortality increases should it progress to a necrotizing process. Here, we present the case of a patient simultaneously using two drugs associated with pancreatitis, which we considered acted synergistically and consequently worsened the patient's outcome.

## Introduction

Acute necrotizing pancreatitis is an important complication of acute pancreatitis that should be actively investigated in patients not improving with standard therapy given its high mortality rates [1,2]. An understanding of the mechanisms leading to necrosis and the potent systemic inflammatory response that follows is required to properly identify and manage cases of necrotizing pancreatitis. With a majority of cases of acute pancreatitis being related to alcoholism and biliary disease, drugs used frequently for conditions with comparatively higher prevalence, such as hydrochlorothiazide and alogliptin, can also precipitate pancreatitis [1,3-4]. Due to their common use, they may however be easily overlooked. In this report, we present a case where these two drugs led to the development of acute pancreatitis, which rapidly became necrotizing and ultimately resulted in a fatal outcome.

## Case presentation

**Figure 1:**
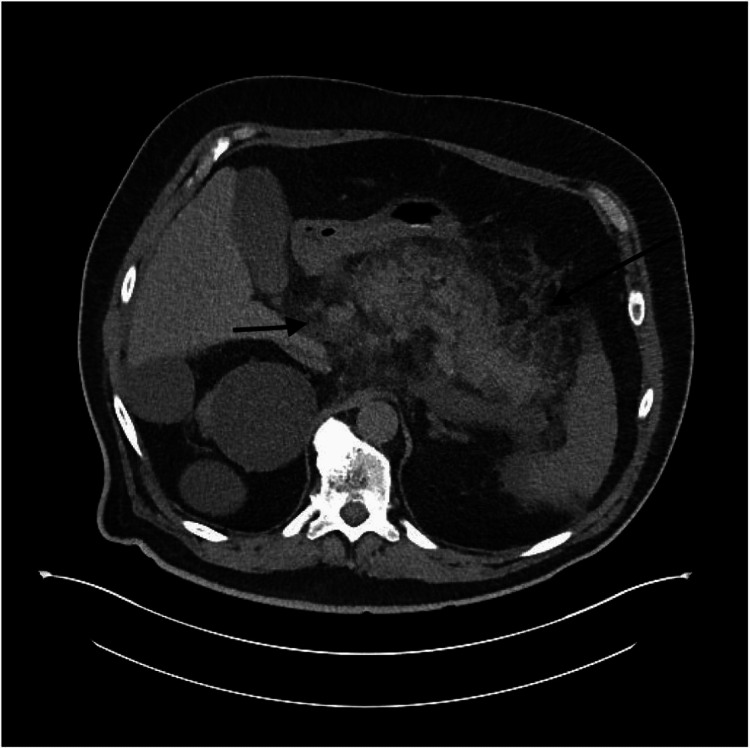
CT abdomen and pelvis showing inflammation in the whole pancreas

**Figure 2:**
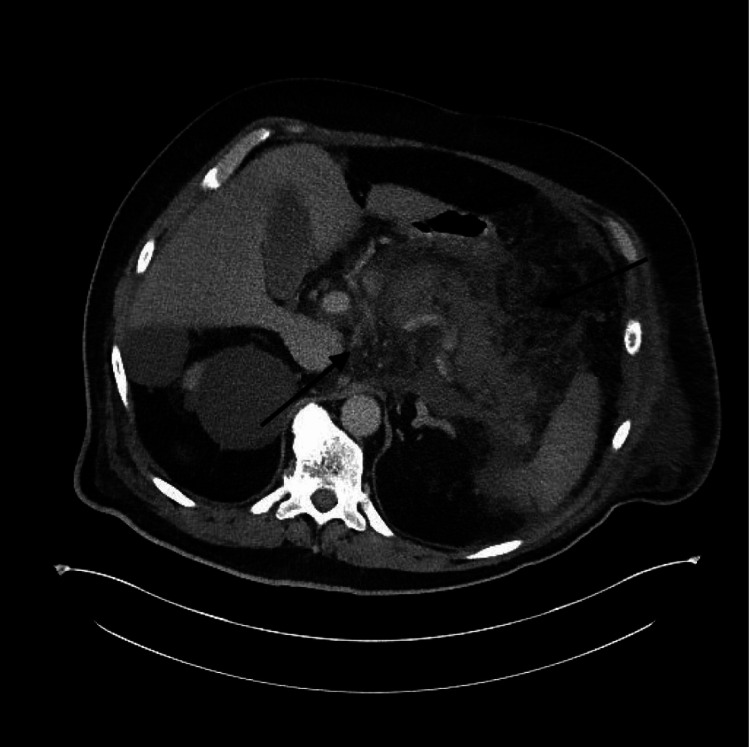
CT abdomen and pelvis shows necrosis in the tail and body of the pancreas

A 70-year-old man with a past medical history of diabetes mellitus and hypertension presented to the emergency department complaining of excruciating epigastric abdominal pain, associated with nausea and vomiting. Home medications included alogliptin taken for the past four years and hydrochlorothiazide taken for around 10 years. The patient reported a remote history of alcohol abuse, having quit 20 years prior. Initial workup was notable for a white cell count of 19.1 k/uL, lipase of 19,144 U/L, creatinine 2.23 mg/dL, blood urea nitrogen (BUN) 53 mg/dL, and triglycerides at 138 mg/dL. Abdominal imaging, including ultrasound and noncontrast computerized tomography (CT), was obtained. Ultrasound showed no evidence of cholelithiasis or cholecystitis. CT showed findings consistent with extensive acute pancreatitis (Figure 1). The patient was admitted to the medical floor and was started on intravenous fluids, a total of 5 liters were administered as well as analgesia with morphine, but eventually, he was transitioned to the intensive care unit due to worsening metabolic acidosis. There, the patient’s condition continued to decline, with progressively worsening renal and respiratory failure. Subsequently, dialysis was initiated and the patient was intubated and placed on mechanical ventilation. A follow-up CT of the abdomen with contrast showed changes now consistent with necrotizing pancreatitis (Figure 2). The patient was initially on cefepime for five days and then transitioned to meropenem, due to worsening status. There were no drainable collections at this time. Surgical intervention was not pursued due to the patient’s critically ill state. The patient’s condition continued to worsen after 12 days of treatment. Upon discussion of the patient's condition and poor prognosis, the patient’s code status was changed to do not resuscitate. He subsequently succumbed to his illness.

## Discussion

A rare, but severe, side effect of using both gliptins and thiazide diuretics separately is acute pancreatitis, which can eventually progress to necrotizing pancreatitis. It was seen in a retrospective study that 3.6% of acute pancreatitis cases were due to drugs, and eight cases out of the 31 (25.8% ) were due to hydrochlorothiazide [1]. Additionally, in the EXAMINE trial, acute pancreatitis was reported in 10 (0.4%) of patients treated with alogliptin and in seven (0.3%) of patients treated with placebo [5-8]. Both of these medications are Class II drugs [5,9]. Class II drugs are described as drugs that have at least 10 reported cases of pancreatitis and there is a consistent latency in 75% or more of the reported cases [5,10-12]. It has been described that these class II drugs have an idiosyncratic reaction that might be related to the accumulation of toxic metabolites, which in 75% of the cases develop into pancreatitis over months to years [5,10-11].

In our patient, the etiology of the necrotizing pancreatitis was multifactorial, the toxicity of the two medications (hydrochlorothiazide and alogliptin) combined and the past social history of alcohol abuse. Necrotizing pancreatitis is diagnosed when more than 30% of the pancreas is necrosed, it has a mortality rate of 5-10% when sterile and 20-30% when infected [2]. Locally, the development of necrosis, especially if it becomes infected accounts for high mortality, but systemic inflammatory response syndrome (SIRS) more than 48 hours and multiple organ failure (renal, respiratory, and/or cardiovascular) following necrosis further increase the risk of fatal outcome [6,10]. 

Since admission, our patient was oliguric with elevated BUN and creatinine, which progressed into kidney failure, requiring dialysis on days 3-4 of admission. Hemodialysis has proven to improve the removal of cytokines and auto-digestive enzymes. In our patient's case, it was not helpful, as he had already developed necrotizing pancreatitis [3]. For this patient who presented with signs of kidney failure, metabolic acidosis could have benefited from early hemodialysis to potentially remove the excess toxins, as has been described in the literature.

## Conclusions

In instances where patients develop rare side effects from certain drugs, this can lead to fatal outcomes such as necrotizing pancreatitis. In this case, we bring into question if taking two different class II drugs simultaneously increases the incidence of developing pancreatitis. In such cases, would patients warrant more aggressive treatment after their presentation? More studies need to be undertaken in order to describe the possible synergism of drugs and identify such cases faster.
